# Draft genome sequences of selected *Klebsiella* clinical strains from a tertiary hospital in the Philippines

**DOI:** 10.1128/mra.00728-25

**Published:** 2025-08-11

**Authors:** Michael Angelou L. Nada, Aubrey Joy P. Tejada, Joseph B. Ancla, Marel Jan G. Joloro, Ruth Antoinette D. Chin, Mark Christian C. Reterta, Anton Roi G. Collado, Janna Ysabelle O. Casidsid, Arra B. Asejo, Rommel J. Gestuveo

**Affiliations:** 1 Department of Science and Technology, Industrial Technology Development Institute 340935, Taguig City, Philippines; 2 Department of Science and Technology, S&T Fellows Program, Taguig City, Philippines; University of Pittsburgh School of Medicine, Pittsburgh, Pennsylvania, USA

**Keywords:** antimicrobial resistance, multidrug-resistant bacteria, draft genome, mobile genetic elements, plasmid, prophage, *Klebsiella pneumoniae*

## Abstract

We reported six *Klebsiella* draft genomes isolated from various clinical samples. These isolates are resistant to various drug classes and harbor at least one β-lactamase gene, plasmid, and prophage region. The results could aid public health strategies and programs in preventing and controlling the spread of high-risk pathogens.

## ANNOUNCEMENT

Antimicrobial resistance (AMR) is a global public health concern. As bacteria recurrently acquire resistance to antibiotics, AMR prevention, diagnosis, treatment, and epidemiology of ESKAPE pathogen group were among the top research priorities set by World Health Organization (1, 2). The *Klebsiella pneumoniae* species complex (KpSC) is responsible for most antimicrobial-resistant infections in the Philippines. In fact, local outbreaks of carbapenem-resistant *K. pneumoniae* have been recorded in selected hospitals, leading to the implementation of stringent monitoring and infection control (3, 4). The genomic resources presented here can be used to investigate the mechanisms underlying antibiotic resistance and the epidemiology of key bacterial pathogens. This is particularly important in low- and middle-income countries (LMIC) where there are high rates of antibiotic resistance, and the capacity for genomic surveillance is being developed.

Six isolates from various clinical specimens (Table 1) were provided by the *Ospital ng Maynila* Medical Center (OMMC) and sub-cultured in Luria-Bertani (LB) agar plates. After 24 h, single colonies were inoculated into 10 mL LB broth and incubated at 37°C. One mL of 18-h-old culture was collected and centrifuged (12,000 rpm, 15 min) to pellet bacterial cells. Genomic DNA was extracted using DNeasy Blood and Tissue kit (Qiagen). Sequencing libraries were prepared using Illumina DNA prep kit (DNA input: 100–500 ng; 30 uL) and sequenced (Illumina MiSeq Reagent Kit v3; 15 pM loading concentration; 2 × 301 bp paired-end chemistry) on Illumina MiSeq platform, generating 11,230,544 sequences (average: ~1,871,758 reads per sample). Read quality was assessed using FastQC v0.11.9 (5) and trimmed (IlluminaClip:TruSeq3-PE-2.fa:2:28:10 SlidingWindow:4:28 Leading:28 Trailing:28 Headcrop:20 MinLen:100) with Trimmomatic v0.39 (6). Reads were *de novo* assembled using SPAdes v3.15.5 (7). Scaffolding and gap filling were done using RagTag v2.1.0 (8). Genome quality, completeness, and coverage were determined using Quast v5.2.0 (9), CheckM v1.2.3 (10), BBMap v39.06 (11), and SAMtools v1.19.2 (12), respectively. Draft genomes were annotated using NCBI Prokaryotic Genome Annotation Pipeline v6.9 (13). Default parameters were used for all programs.

**TABLE 1:** Genomic features and antibiotic resistance profile of selected *Klebsiella* clinical isolates^*a*^

Categories	Clinical isolate / strain					
	OMCS-21- 1430	OMCS-22- 1557	OMCS-22- 1561	OMCS-22- 1963	OMCS-22- 1274	OMCS-22- 1797
**Isolate information**						
Specimen	Sputum	Urine	Wound discharge	Urine	Urine	Sputum
Culture condition	Blood Agar Plates and MacConkey Agar, 18–24 h, 35–37°C	Blood Agar Plates and MacConkey Agar, 18–24 h, 35–37°C	Blood Agar Plates and MacConkey Agar, 18–24 h, 35–37°C	Blood Agar Plates and MacConkey Agar, 18–24 h, 35–37°C	Blood Agar Plates and MacConkey Agar, 18–24 h, 35–37°C	Blood Agar Plates and MacConkey Agar, 18–24 h, 35–37°C
Antibiotic resistance profile^*b*^	Penicillin, Lincosamide	Penicillin, Cephalosporin, Aminoglycosides, Fluoroquinolones, Sulfonamide + Trimethoprim	Penicillin, Cephalosporin, β-lactams-β-lactamase Inhibitors, Tetracycline, Sulfonamide + Trimethoprim	Penicillin, Carbapenem, Tetracycline	Penicillin	Penicillin, Cephalosporin, Carbapenem, Monobactam, β-lactams-β-lactamase Inhibitors, Sulfonamide + Trimethoprim
**Genome features**						
Raw reads	1,841,851	1,924,540	1,894,706	1,623,814	2,045,156	1,900,477
Trimmed reads	1,081,281	1,103,186	1,098,351	945,999	1,206,506	1,545,155
Genome size (bp)	5,303,885	5,306,195	5,622,561	6,130,390	5,527,980	5,607,542
No of contigs	40	29	71	77	25	32
GC content (%)	57.42	57.42	57.02	57.03	57.28	57.04
Largest contig (bp)	3,601,677	5,137,435	5,268,659	5,423,600	5,243,698	5,259,322
N50^*c*^	3,601,677	5,137,435	5,268,659	5,423,600	5,243,698	5,259,322
N90^*d*^	354,122	5,137,435	5,268,659	112,740	5,243,698	5,259,322
L50^*e*^	1	1	1	1	1	1
Mean coverage (X)	85	87	82	66	92	123
Completeness (%)	100	100	100	100	100	100
Contamination (%)	0.04	0.03	0.63	0.97	1.20	0.45
Total gene	5,258	5,262	5,681	6,132	5,411	5,532
Total CDS	5,153	5,157	5,575	6,022	5,306	5,431
Functional proteins	5,050	5,054	5,379	5,881	5,161	5,283
Hypothetical proteins	103	103	196	141	145	148
CRISPR array	0	0	0	0	1	1
Genes (RNA)	105	105	106	110	105	101
ncRNA	10	10	12	14	9	8
tRNA	78	79	80	83	82	80
tmRNA	1	1	1	1	1	1
MLST	ST661	ST661	ST3838	ST196	ST23	ST39
wzi alles	wzi8	wzi8	wzi173	wzi97	wzi1	wzi62
Capsule (K) serotype	K8	K8	Unknown (KL155)	Capsule null	K1	K62
O antigen (LPS) serotype	O2afg	O2afg	O13	O3/O3a	O1ab	O1ab
Virulence score^*f*^	0	0	0	0	5	1
Resistant score^*g*^	0	0	0	0	0	3
**Antibiotic resistance genes (ARGs)** ^***h***^						
Aminoglycoside	aac (3)-IId, aadA2, aph3-Ia, strA, strB	aac (3)-IId, aadA2, aph3-Ia, strA, strB	–	aac (3)-IId, aac(6')-Ib-cr, aadA5	–	aph3-Ia, strA, strB
Colistin	–	–	–	–	–	MCR-8.2
Fosfomycin	–	–	–	–	–	–
Fluoroquinolone	qnrS1	qnrS1	–	qnrB1	–	qnrS1
Glycopeptide	–	–	–	–	–	–
Macrolide	Mrx, mphA	Mrx, mphA	–	Mrx, mphA	–	erm(42)
Phenicol	–	–	–	CatB4	–	catII.2
Rifampin	–	–	–	–	–	–
Sulfonamide	sul1, sul2	sul1, sul2	–	sul1	–	sul2
Tetracycline	–	–	–	tet(D)	–	–
Tigecycline	–	–	–	–	–	–
Trimethoprim	dfrA12	dfrA12	–	dfrA17	–	–
β-lactamase	–	–	–	OXA-1	–	–
β-lactamase with resistance to β-lactamase inhibitors	–	–	–	–	–	–
Extended-spectrum β-lactamases (ESBL)	–	–	–	–	–	CTX-M-15
ESBL with resistance to β-lactamase inhibitors	–	–	–	–	–	–
Carbapenemase	–	–	–	–	–	OXA-48
Penicillin	–	SHV-27	SHV-110	OKP-A-5	SHV-11	SHV-11
**Virulence determinants** ^***h***^						
Yersiniabactin	–	–	–	–	ybt1, ICEKp10	ybt15, ICEKp11
Colibactin	–	–	–	–	clb 2	-
Aerobactin	–	–	–	–	iuc 1	-
Salmochelin	–	–	–	–	iro 1	-
RmpADC	–	–	–	–	rmp 1, KpVP-1	-
rmpA2	–	–	–	–	rmpA2_6-60%	-
**Taxonomic assignment**						
Identification	*Klebsiella pneumoniae*	*Klebsiella pneumoniae*	*Klebsiella pneumoniae*	*Klebsiella quasipneumoniae subsp. quasipneumoniae*	*Klebsiella pneumoniae*	*Klebsiella pneumoniae*
Average Nucleotide Identity (%)	99.09	98.79	98.86	98.89	98.87	98.97
Accession no. of closest relative	GCF_000742135.1	GCF_000742135.1	GCF_000742135.1	GCF_020525925.1	GCF_000742135.1	GCF_000742135.1
**Mobile genetic elements (MGEs)**						
Plasmid number / type^*i*^	One conjugative; One non-mobilizable	One conjugative; One non-mobilizable	Two non-mobilizable; Two mobilizable	Two conjugative; Two non-mobilizable	One non-mobilizable	Two conjugative; Two non-mobilizable; Four mobilizable
Notable plasmids	IncFIB + IncFII	IncFIB + IncFII	Novel type	IncHI1B + IncFIB,	IncFIB + HI1B	IncX1/X3, Col156, IncQ1
Prophage region^*j*^	Three prophage regions	Three prophage regions	Three prophage regions	Three prophage regions	Two prophage regions	No prophage region
GenBank accession number	JBJOUR000000000	JBJOUQ000000000	BJOUP000000000	JBJOUO000000000	JBJOUN000000000	JBJOUM000000000

^*a*^ Clinical isolates were fully de-identified and used exclusively for genomic analysis with permission (sample transfer) from Ospital ng Maynila Medical Center (OMMC). No patient data were accessed. Ethical approval was not required.

^*b*^ Antibiotic susceptibility testing (AST) was done by the Department of Pathology and Laboratory Medicine (OMMC) using Thermo Scientific Sensititre OptiRead Automated Fluorometric Plate Reading System following the 2023 Clinical and Laboratory Standards Institute (CLSI M100 33^rd ^Edition) guidelines*.*

^*c*^ N50 = the contig length by which half of the contigs in the genome assembly is equal to or larger than the N50 contig size.

^*d*^ N90 = shortest contig length needed to cover at least 90% of the total genome size.

^*e*^ L50 = the smallest number of contigs needed to cover approximately half of the total genome size.

^*f*^ Virulence score: 0 = negative for all of yersiniabactin (ybt), colibactin (clb), aerobactin (iuc); 1 = yersiniabactin only; 2 = yersiniabactin and colibactin (or colibactin only); 3 = aerobactin (without yersiniabactin or colibactin); 4 = aerobactin with yersiniabactin (without colibactin); 5 = yersiniabactin, colibactin, and aerobactin (and/or salmochelin)*.*

^*g*^ Resistant score: 0 = no ESBL, no carbapenemase (regardless of colistin resistance); 1 = ESBL, no carbapenemase (regardless of colistin resistance); 2 = Carbapenemase without colistin resistance (regardless of ESBL genes or OmpK mutations); 3 = Carbapenemase with colistin resistance (regardless of ESBL genes or OmpK mutations).

^*h*^ "–” indicates the absence of genes or undetected in bacterial genomes.

^*i*^ Plasmid mobility is assigned based on the presence of relaxase (mobilizable) and/or MPF proteins (conjugative) or the absence of both (non-mobilizable)*.*

^*j*^ Only sequences with “provirus” topology and virus score of >0.95 were used. Prophage genome completeness ranges from 95% to 100% and was classified as “high-quality” genomes using checkV v1.0.1*. *

Genome size ranges from 5.30 to 6.13 Mbp (mean coverage: 66–123×) with GC content of 57.02% to 57.42%. High-quality draft genomes (100% complete; <1.2% contamination) were composed of 25 to 77 contigs (N50 >3,600 Kbp) and 5,153-6,022 protein-coding sequences. Five isolates were classified as *K. pneumoniae* (98.79%–99.09% ANI) using GTDB-Tk v2.4.0 (14, 15); with one isolate (OMCS-22-1963) identified as *K. quasipneumoniae subsp. quasipneumoniae* (98.89% ANI). The complete genome features were presented in Table 1.

**Fig 1:**
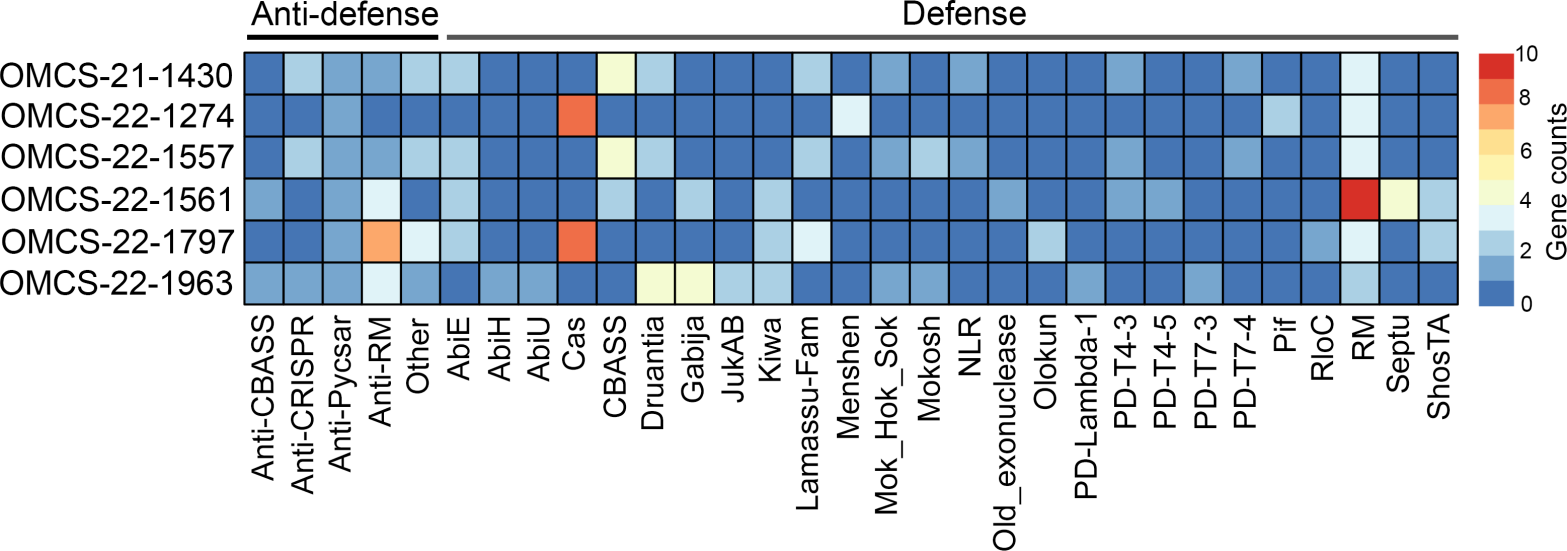
Defense and anti-defense system in *Klebsiella* clinical strains. Color represents the number of genes that encode for defense and anti-defense proteins in *Klebsiella* clinical isolates (blue = low, red = high).

AMR profiling using Kleborate v3.1.2 (16) and phenotypic testing confirmed that these isolates are resistant to a total of nine drug classes (Table 1). Five isolates acquired resistance to aminoglycoside, fluoroquinolone, macrolide, sulfonamide, phenicol, trimethoprim, and penicillin. Notably, OMCS-22-1797 harbors the *bla_CTX-M-15_* and MCR-8.2 genes, conferring extended-spectrum β-lactamase (ESBL) and colistin resistance. We identified several plasmids, prophages, defense, and anti-defense systems in bacterial genomes (Table 1; Fig. 1) using MOB-suite v3.1.9 (17), geNomad v1.8.1 (18), and DefenseFinder v1.3.0 (19). This information on mobile genetic elements (MGEs) could guide local efforts to manage *Klebsiella* MDR infections and fine-tune predictive models to better understand phage-host interactions.

## Data Availability

The raw sequences generated in this study were deposited in NCBI Sequence Read Archive (SRA) under Bioproject PRJNA1190650. The draft genome assemblies are publicly available in DDBJ/ENA/GenBank database and can be accessed using the accession numbers listed in Table 1.
